# Cryoprotective Role of Soybean Oligosaccharides in Frozen Dough: Enhanced Gluten Stability and Bread Quality

**DOI:** 10.3390/foods15162878

**Published:** 2026-08-18

**Authors:** Yongxin Gao, Yen Nee Tan, Mei Kying Ong, Mingshuang Wang, Haitao Sun, Xinru Shao, Shyan Yea Chay

**Affiliations:** 1 Department of Agricultural and Food Science, Faculty of Science, Universiti Tunku Abdul Rahman, Kampar 31900, Malaysia; xinxin_sp0601@163.com (Y.G.); chaysy@utar.edu.my (S.Y.C.); 2 School of Public Health, Jilin Medical University, No. 5 Jilin Street, Jilin 132013, China; wms0917@126.com (M.W.); shtjlu@126.com (H.S.); shaoxinru@126.com (X.S.)

**Keywords:** water distribution, rheological properties, protein secondary structure, ice recrystallization, texture profile analysis

## Abstract

Frozen dough technology faces persistent quality deterioration due to ice crystal formation and gluten network disruption during storage. This study investigated the cryoprotective effects of soybean oligosaccharides (SBOS) on frozen dough quality. Wheat flour doughs supplemented with 0%, 0.2%, 0.5%, and 0.8% SBOS (*w*/*w* flour basis) were rapidly frozen at −40 °C, stored at −18 °C for 0, 2, and 4 weeks, and subsequently analyzed for fermentation performance, rheological properties, water distribution, protein secondary structure, microstructure, and bread quality. Results demonstrated that SBOS supplementation significantly attenuated freeze-induced quality deterioration. SBOS effectively inhibited bound-to-free water conversion, preserved gluten network integrity, and maintained higher α-helix content in gluten proteins. Dough with 0.5% SBOS exhibited superior fermentation volume, dynamic rheological properties, and microstructural stability throughout frozen storage. Corresponding bread showed increased specific volume, reduced hardness (15.27% decrease), and improved springiness, cohesiveness, and pore uniformity. Correlation analysis confirmed that SBOS concentration was not linearly associated with quality improvements, with 0.5% identified as the optimal dosage. These findings establish that SBOS is an effective, natural, clean-label cryoprotectant for frozen dough under the tested conditions, offering significant quality preservation benefits for the frozen bakery industry. Future studies are warranted to investigate potential additional functionalities.

## 1. Introduction

Bread is one of the most widely consumed staple foods worldwide and plays an important role in daily nutrition and the global bakery industry. Increasing consumer demand for fresh, convenient, and high-quality bakery products has promoted the rapid development of frozen dough technology. Among various bakery products, white bread is commonly used as a representative model for evaluating frozen dough quality because its quality is highly dependent on dough fermentation and gluten network integrity. Frozen dough technology has become an integral part of the modern baking industry since its development in the mid-20th century. By storing and transporting shaped dough under low-temperature conditions, this technology enables standardized and large-scale bread production, decouples production from retail timing, reduces labor costs, extends product shelf life, and meets consumer demand for freshly baked products [1]. Furthermore, frozen dough technology plays a vital role in ensuring food supply stability, reducing food waste at the retail level, and providing economic benefits to both producers and consumers through improved logistics efficiency [2]. However, the quality deterioration of frozen dough during frozen storage remains a critical technological bottleneck limiting its widespread application [3].

The deterioration mechanisms of frozen dough quality are multifactorial. During freezing and frozen storage, the formation and recrystallization of ice crystals within the dough matrix cause physical damage to the gluten network, leading to structural loosening and discontinuity [4]. Simultaneously, ice crystal formation disrupts yeast cell membranes, reducing yeast viability and subsequently impairing dough fermentation capacity and gas retention. Moisture migration and redistribution during frozen storage alter the water status within the dough, exacerbating the hydration imbalance of gluten proteins [5]. Furthermore, changes in protein secondary structure, particularly the decrease in α-helix content and increase in β-sheet content [6], reflect conformational deterioration of gluten proteins, directly affecting the viscoelasticity and processing performance of the dough. Collectively, these factors result in reduced bread specific volume, increased crumb hardness, and non-uniform pore structure, severely compromising sensory quality and consumer acceptance [7,8].

To ensure frozen dough quality, various strategies have been developed to mitigate freeze-induced damage. Conventional cryoprotectants such as sugars, hydrocolloids, emulsifiers, and phosphates have been widely used to regulate ice crystal growth, stabilize water distribution, and protect protein structure [9]. Among these, carbohydrates have garnered considerable attention due to their operational simplicity and significant effectiveness. The cryoprotective efficacy of starch-based compounds, notably certain hydroxyethyl starch, has been established as a beneficial supplement to dimethylsulfoxide-based cryopreservation protocols, though the optimal admixture ratios remain to be systematically investigated [10]. Research by Su et al. [11] has confirmed that sodium alginate-trehalose interactions confer enhanced thermal stability to the ternary complex. Progressive increases in complex concentration accelerated dough passage through the critical ice-formation zone, generating smaller, uniform ice crystals. The 0.8% addition level optimized dough quality, as evidenced by increased bound water retention and improved hardness, springiness, and cohesiveness. Su et al. [12] demonstrated that carboxymethyl cellulose (CMC) effectively enhanced frozen dough quality during storage by stabilizing starch structure and ensuring uniform moisture distribution. Su et al. [12] achieved optimal results with 3% CMC, which maximized cohesiveness and water retention. Hardness and chewiness peaked at 3% CMC in wheat dough and at 4% CMC in corn multigrain dough, respectively. However, most of these sugars and their derivatives are utilized as food additives, rendering them less appealing to consumers. The public exhibits a strong preference for compounds naturally present in plants. Consequently, the identification of natural, harmless, plant-derived cryoprotectants has remained a persistent research objective [13].

Although plant-derived cryoprotectants exhibit promising antifreeze properties, their structural characteristics, cryoprotective mechanisms, and optimal application conditions vary considerably depending on the source material [14]. Soybean oligosaccharides (SBOS) are functional carbohydrates extracted from soybeans, primarily composed of raffinose, stachyose, and sucrose [15]. Due to its moderate sweetness and high viscosity, SBOS is widely utilized as a food additive to enhance flavor and mouthfeel [16]. Additionally, the health-promoting functions of SBOS have attracted increasing attention. Historically regarded as an antinutritional factor due to its association with gastrointestinal discomfort, including bloating and diarrhea [17], recent research has revealed its physiological roles in modulating gut microbiota, promoting nutrient and mineral absorption, reducing blood pressure and glucose levels, and enhancing immune function, thereby garnering significant research interest [18]. SBOS exhibits excellent water-holding capacity and prebiotic activity and has been widely applied in dairy products, bakery items, and functional foods [19]. Recent studies have suggested that SBOS, a member of oligosaccharides, can suppress ice recrystallization, maintain the conformational stability of proteins, and improve the textural properties of food [20,21]. As a class of carbohydrate molecules, oligosaccharides have attracted substantial research attention for their utilization as cryoprotectants, which provides a solid research basis for exploring the functional performance of SBOS. The unique glycosidic bond structure of SBOS enables interactions with water molecules and protein surfaces, potentially modulating ice crystal growth and preserving protein structural integrity during freezing [22,23]. Compared with other cryoprotectants, SBOS offers the advantages of natural origin, commercial availability, and established safety for food applications. However, research on the application of SBOS in frozen dough systems remains limited. To date, systematic investigations into the effects of SBOS on gluten protein structure, moisture migration behavior, fermentation performance, and subsequent baking quality during frozen storage are scarce, and the underlying mechanisms and optimal dosage remain unclear.

Therefore, this study aimed to systematically evaluate the effects of different SBOS concentrations (0%, 0.2%, 0.5%, and 0.8%) on the fermentation characteristics, dynamic rheological properties, water distribution, protein secondary structure, and microstructure of frozen dough after 0, 2, and 4 weeks of frozen storage. Additionally, the specific volume, textural properties, and pore structure of the resulting bread were assessed. The objectives were to elucidate the potential mechanisms by which SBOS improves the freezing stability of dough, determine the optimal SBOS concentration, and provide a theoretical foundation for the application of SBOS in the frozen dough industry.

## 2. Materials and Methods

### 2.1. Materials and Chemicals

Wheat flour (protein content: 12.2%, moisture content: 11.89%, ash content: 0.48%, wet gluten content: 30.5%) was obtained from Yihai Kerry Arawana Holdings Co., Ltd. (Shanghai, China). SBOS (purity ≥ 90%, composition: raffinose 22%, stachyose 48%, sucrose 15%, other oligosaccharides 15%) was purchased from Henan Wanbang Industrial Co., Ltd. (Zhengzhou, China). Potassium bromide (spectroscopic grade, ≥99.5%) and sodium chloride (analytical grade, ≥99.5%) were obtained from Shanghai Macklin Biochemical Technology Co., Ltd. (Shanghai, China). Active dry yeast (Saccharomyces cerevisiae, viability ≥ 10^10^ CFU/g) was purchased from Angie’s Yeast Co. (Yichang, China). Unsalted butter (fat content 82%, moisture content 16%) from Anchor brand was obtained from Fonterra Co-operative Group Limited (Auckland, New Zealand). White granulated sugar (sucrose content ≥ 99.5%) and table salt (sodium chloride content ≥ 99.0%) were purchased from a local supermarket. Deionized water was used for all experiments.

### 2.2. Preparation of Frozen Dough

The preparation method for frozen dough was adapted from Li et al. [24] and Sheikholeslami et al. [25] with adaptations. The direct dough method was employed for dough preparation. The basic formulation comprised 600 g wheat flour, 48 g sugar, 4.8 g salt, 9 g yeast (active dry yeast rehydrated in 30 mL warm water at 35 °C for 10 min prior to use), and 24 g butter (softened at room temperature). Wheat flour and water were mixed at a ratio of 5:3 (*w*/*w*) in a dough mixer (SM2-10, Sinmag, Wuxi, China) at low speed (60 rpm) for 3 min, followed by medium speed (120 rpm) for 5 min until a homogeneous dough was formed. The butter was added after 2 min of mixing. The resulting dough was divided into 150 g portions, shaped into round balls, and wrapped in polyethylene film. The dough samples were subjected to rapid freezing at −40 °C for 2 h in a blast freezer (NX-12C-45, Nanxia, Foshan, China), followed by frozen storage at −18 °C in a freezer (BCD-215SEBB, Haier, Qingdao, China) for 0, 2, and 4 weeks. The freezing storage durations were set at 0, 2, and 4 weeks. As a cold-chain bakery item, frozen dough is produced centrally and shipped to retail points where it is baked fresh for consumers. This distribution model results in frequent replenishment cycles (every 3–5 days) rather than extended retail storage. Therefore, an experimental storage duration of one month adequately represents typical supply chain conditions and allows observation of progressive quality changes. However, it is acknowledged that longer storage periods (e.g., 3–6 months) may be relevant for certain industrial applications, and this represents a limitation of the present study. Future research should investigate the protective effects of SBOS over extended storage periods to fully assess its long-term cryoprotective efficacy. After 0, 2, and 4 weeks of frozen storage, the dough samples were thawed at 4 °C in a refrigerator prior to subsequent analysis. Subsequent analyses were performed. SBOS was incorporated at concentrations of 0.2%, 0.5%, and 0.8% (*w*/*w*, based on flour weight), with the non-supplemented dough serving as the control.

### 2.3. Preparation of Bread

The bread prepared in this study was white bread produced using the straight dough method. The bread preparation method proposed by Zhang et al. [26] was adopted with minor modifications to meet the experimental requirements. Frozen dough samples were thawed at 4 °C in a refrigerator for 8 h. The thawed dough pieces were then placed in a fermentation chamber (SPC-40FP, Sinmag, Wuxi, China) maintained at 35 °C and 85% relative humidity for 90 min. Subsequently, the proofed dough was baked in a convection oven (SJ3-921, Sinmag, Wuxi, China) at 170 °C for 20 min. Each batch consisted of three dough samples baked simultaneously in the same oven. Three independent batches were performed for each experimental condition. After baking, bread samples were cooled at ambient temperature (25 ± 1 °C) for 2 h before quality parameter measurements. The same oven was used for all baking experiments to ensure consistent heat distribution.

### 2.4. Fermentation Volume

The dough fermentability was determined according to AACC International Method 10-05.01 with modifications described. Frozen dough was thawed to ambient temperature. A 10 g sample was placed into a 50 mL graduated cylinder, and the initial volume was recorded. The cylinder was then incubated in a fermentation chamber (SPC-40FP, Sinmag, Wuxi, China) maintained at 35 °C and 85% relative humidity. Dough volume was measured at 30 min intervals until two consecutive readings remained constant.

### 2.5. Dynamic Rheological Analysis

Dynamic rheological measurements were performed on a Kinexus Lab+ rheometer (Malvern Panalytical, Malvern, UK) according to the method described by Dufour et al. [27] and Meziani et al. [28]. The dough was positioned between two parallel plates (40 mm diameter, 1 mm gap). Excess sample was trimmed, and the exposed edges were coated with silicone oil to prevent moisture loss. The measurement geometry was lowered to the preset gap, and the oscillatory test was initiated. The experimental parameters were as follows: strain amplitude of 0.1%, temperature of 25 °C, and frequency sweep range of 0.1–50 Hz.

### 2.6. Water Distribution Analysis (LF-NMR)

Transverse relaxation time (T_2_) measurements were performed on a benchtop Low-Field Nuclear Magnetic Resonance (LF-NMR) imaging analyzer (NM120-025V-I, Niumag Electronic Technology Co., Ltd., Shanghai, China). The CPMG pulse sequence was employed following the procedure of Jiang et al. [29] and Li et al. [30]. Frozen dough samples with varying storage periods were thawed at ambient temperature and promptly sealed in polyethylene film to minimize dehydration. Data acquisition was conducted at a proton resonance frequency of 20 MHz with the following optimized parameters: P90 = 7.00 μs, P180 = 17.00 μs, TD = 29,020, SW = 100 kHz, TW = 5000 ms, NS = 32, RG_1_ = 20, DRG_1_ = 3, and PRG = 0.

### 2.7. Secondary Structure Analysis (FTIR)

Freeze-dried dough powder was thoroughly blended with potassium bromide at a mass ratio of 1:100, followed by fine grinding in an agate mortar to obtain a homogeneous mixture. The resulting translucent pellets were subjected to Fourier transform infrared spectroscopy (FTIR) for protein secondary structure characterization. Instrumental parameters were configured as follows: 32 scans were accumulated at a resolution of 4 cm^−1^, covering the spectral range of 400–4000 cm^−1^. Subsequent data manipulation was performed using OMNIC 9 (Thermo Fisher Scientific, Waltham, MA, USA) and PeakFit V4.12 (Cabit Information Technology Co., Ltd., Shanghai, China). The amide I region (1600–1700 cm^−1^) was extracted and processed through baseline correction, Gaussian smoothing, and curve deconvolution to generate the secondary structure distribution profile. Peak assignments were made by correlating observed wavenumbers with established literature references, enabling quantitative determination of the relative abundance of each structural component [31].

### 2.8. Scanning Electron Microscopy (SEM)

The frozen dough morphology was observed using a scanning electron microscope (SEM, JSM-6701F, JEOL, Tokyo, Japan). Samples were immobilized on conductive tape and coated with gold, and then micrographs were captured at an accelerating voltage of 4.0 kV.

### 2.9. Specific Volume of Bread

The specific volume of bread was determined according to the reported method of Zhang et al. [32] with minor modifications. The bread was cooled at room temperature for 2 h, and its weight (g) was recorded using an electronic balance. The volume (mL) of the bread was determined using the millet replacement method with a JMTY bread volume analyzer (Daji Optoelectronic Instrument Co., Ltd., Hangzhou, China). The specific volume of bread was calculated using Formula (1):

(1) $$\mathrm{Specific}\;\mathrm{volume}\;(\mathrm{mL}/g)=\frac{\mathrm{Volume}\;\mathrm{of}\;\mathrm{bread}\;(\mathrm{mL})}{\mathrm{Weight}\;(g)}$$

The average of the three replicates was recorded.

### 2.10. Texture Analysis of Bread (TPA)

The texture of the bread was assessed using a TA-XT Plus Texture Analyzer (Stable Micro Systems Co., Ltd., Surrey, UK) equipped with a cylindrical probe P/2, following a method outlined by Liu et al. [8] with minor modifications. After baking and cooling to room temperature, the bread was sliced to a thickness of 2 cm using a slicer. Slices of uniform size were taken from the central portion for subsequent analysis. A texture profile analysis was applied to the samples with a compression rate of 60%, pre-test speed of 2.0 mm/s, test speed of 1.0 mm/s, post-test speed of 2.0 mm/s, and trigger force of 5.0 g. The time interval between two compressions was 5 s. The hardness, chewiness, springiness, cohesiveness, and resilience of the bread were calculated according to the time-force deformation curve of texture profile analysis. For each experimental condition, three independently baked bread loaves were prepared. After cooling, one central slice (2 cm thickness) was taken from each loaf, and the three slices were measured individually on the texture analyzer. The measurements were averaged to obtain the final texture data for that condition.

### 2.11. Internal Texture Structure of Bread

A central region of 2.14 cm * 2.14 cm was excised from each bread slice. Image acquisition and analysis were performed using ImageJ software 1.53t (National Institutes of Health, Bethesda, MD, USA). Images were captured at a resolution of 600 dpi and converted to 8-bit grayscale for binary threshold processing. Pore density (number of pores per unit area) and average pore area were subsequently quantified through image segmentation and particle analysis using ImageJ software, following the method described by Hong et al. [33].

### 2.12. Data Analysis

Data are presented as mean ± standard deviation (SD) based on three independent replicate measurements (*n* = 3). Two-way analysis of variance (ANOVA) was performed using SPSS Statistics 27 (IBM Corp., Armonk, NY, USA) with SBOS concentration (0%, 0.2%, 0.5%, 0.8%) and frozen storage time (0, 2, 4 weeks) as the two independent factors. A significance threshold was defined as *p* < 0.05. Data visualization was performed using Origin 2021 (OriginLab Corp., Northampton, MA, USA) and ImageJ software.

## 3. Results and Discussion

### 3.1. Fermentation Volume of Frozen Dough

**Figure 1:**
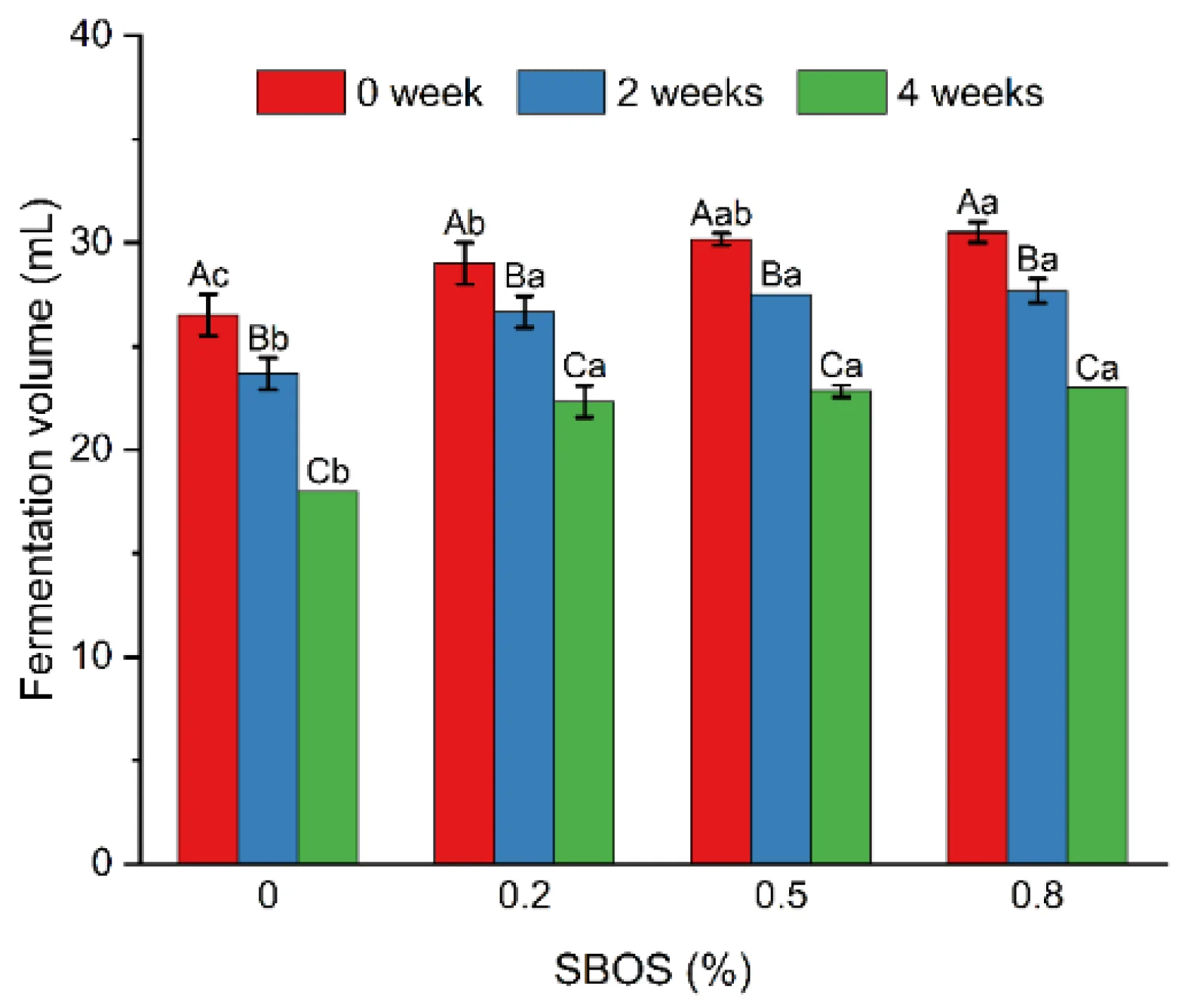
Effects of different SBOS ratios on fermentation characteristics of frozen dough (Lowercase letters indicate significant differences among concentrations at the same freezing time, *p* < 0.05; capital letters indicate significant differences among freezing times at the same concentration, *p* < 0.05).

Dough fermentation volume is an important indicator of baking performance and the quality of the final bread. Under identical processing conditions, a positive correlation exists between dough expansion capacity and final bread quality within a defined range [34]. The volume of dough after fermentation is a critical indicator of its gas retention capacity. Studies by Yang et al. [35] have demonstrated that freezing dough results in reduced fermentation volume and a weakened gluten network structure. As illustrated in Figure 1, prolonged frozen storage significantly reduced dough fermentation volume. SBOS supplementation significantly increased the fermentation volume at 0, 2, and 4 weeks of storage compared with the 0%SBOS group, indicating that SBOS supplementation effectively enhances dough fermentation capacity. Furthermore, Figure 1 shows that the improvement in fermentation volume became more pronounced with extended storage duration, suggesting that the beneficial effect of SBOS on dough fermentability strengthens over time. Regarding dosage, no significant differences were observed among the 0.2%, 0.5%, and 0.8% SBOS treatments, except at week 0, where 0.2% and 0.8% SBOS showed significant variation. These findings demonstrate that even a low dosage of SBOS incorporation into flour can significantly improve the fermentation performance of frozen dough. This result may be due to SBOS incorporation effectively preserving yeast viability and gluten network integrity during frozen storage, thereby maintaining the dough’s ability to trap and retain carbon dioxide during the proofing process. Similarly, studies have shown that the incorporation of various ingredients can affect the fermentative capacity of dough after freezing. For instance, Li et al. [36] found that the combined addition of 0.2–0.6% pectin, 0.2–1% guar gum, and 0.6–1% Arabic gum enhanced the gas retention properties of dough. In summary, SBOS effectively improves fermentation volume caused by frozen storage. However, the mechanism of its effect on yeasts warrants further investigation. Determining the optimal SBOS dosage for industrial frozen dough production remains significant and requires additional experimental research. This will provide data support for enhancing product quality stability and extending shelf life.

### 3.2. Dynamic Rheological Properties of Frozen Dough

**Figure 2:**
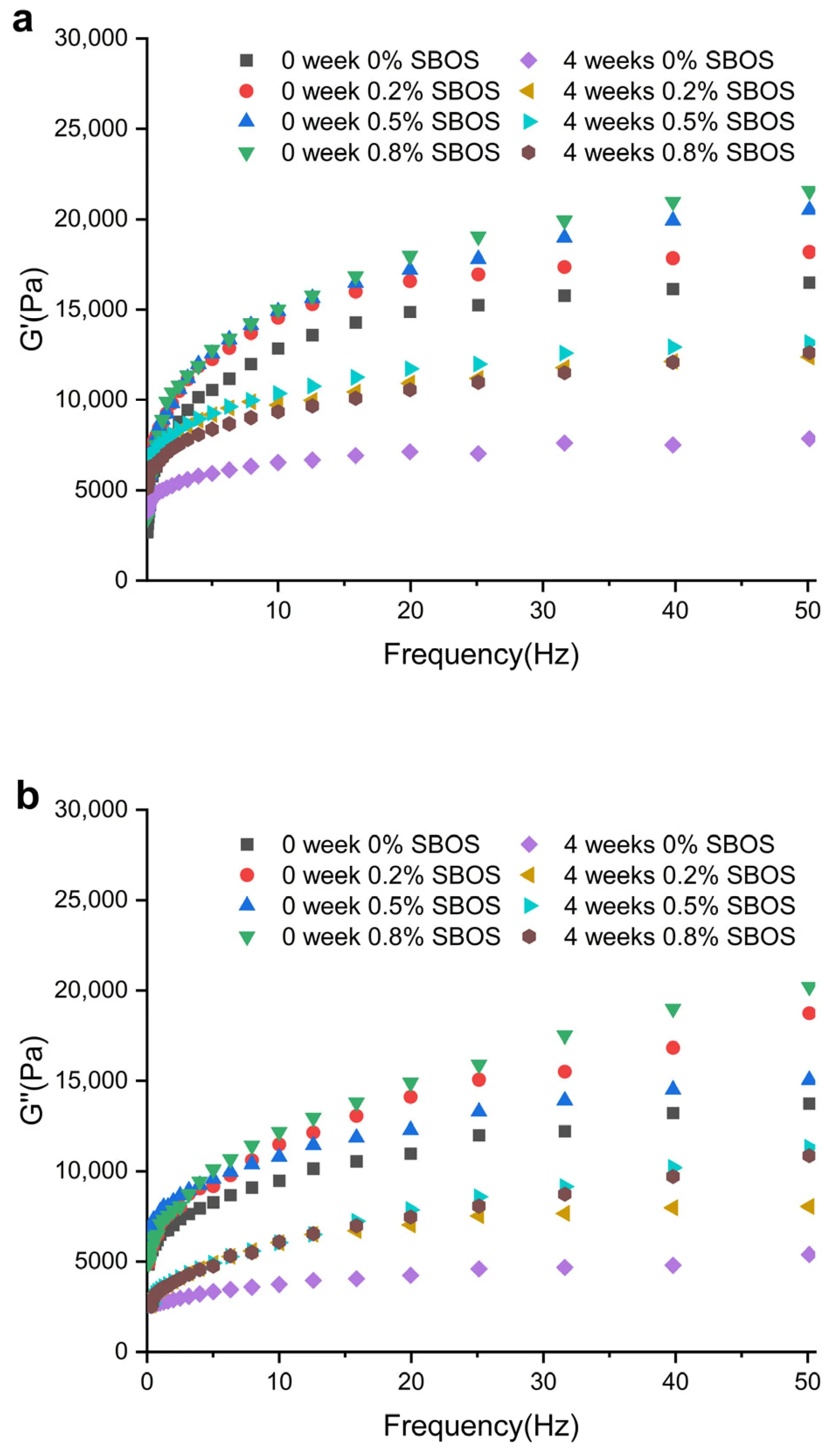
Effects of SBOS additions on the ratios of G′ (**a**) and G″ (**b**).

In rheological characterization of dough systems, the storage modulus (G′) and loss modulus (G″) serve as fundamental indicators of viscoelastic behavior. G′ represents the elastic component and solid-like characteristics of the dough, quantifying the capacity of the gluten network to store deformation energy elastically. An elevated G′ value typically signifies a robust and well-developed gluten network, manifesting as enhanced dough rigidity, greater resistance to deformation, and superior gas-holding capacity during fermentation. Conversely, G″ reflects the viscous component and liquid-like behavior, associated with molecular segment mobility and intermolecular interactions (including protein-protein and protein-starch associations). Higher G″ values indicate increased dough fluidity and extensibility; however, excessive viscous character may compromise the dough’s ability to retain fermentation gases, potentially leading to diminished loaf volume [37]. The dynamic rheological parameters of different dough formulations were investigated, with results illustrated in Figure 2. Figure 2a presents the storage modulus (G′), while Figure 2b shows the loss modulus (G″) across all treatment groups. The rheological properties of dough fundamentally govern the textural and sensory qualities of the resulting baked goods [38]. Within the experimental frequency range, both G′ and G″ increased with frequency, displaying typical viscoelastic behavior. The consistent dominance of G′ over G″ across all samples confirms the predominantly elastic nature of these dough systems.

Frozen storage significantly impacted the rheological profile of the dough samples. The SBOS group exhibited progressively decreasing G′ and G″ values with extended frozen storage, with marked reductions detected at week 4 relative to 0%SBOS dough (week 0). This temporal decline in rheological properties suggests that prolonged frozen storage exerts a detrimental effect on both the elastic and viscous characteristics of the dough system. Over extended freezing periods, the progressive growth of ice crystals induces physical disruption within the dough microstructure, accompanied by non-uniform moisture redistribution between the gluten phase, starch granules, and the aqueous phase. These combined effects weaken the structural integrity of the gluten-starch network, impairing its ability to retain elasticity and resist deformation. Consequently, both the elastic (G′) and viscous (G″) moduli decrease over time, reflecting a general deterioration in dough functionality caused by freeze-induced structural damage. This finding is consistent with the results reported by Yang et al. [39].

However, SBOS incorporation demonstrably attenuated this deterioration. At each frozen storage time, SBOS-containing doughs maintained significantly higher G′ values compared with 0%SBOS samples, with this protective effect persisting through week 4 (Figure 2a). This suggests that SBOS enhances the freeze–thaw tolerance of dough, presumably by stabilizing the gluten protein network against the mechanical stresses imposed by ice crystal formation and recrystallization. The sustained elevation of G′ indicates preserved structural integrity of the dough matrix, which is conducive to maintaining bread volume and crumb structure in the final product.

### 3.3. Water Distribution in Frozen Dough

**Figure 3:**
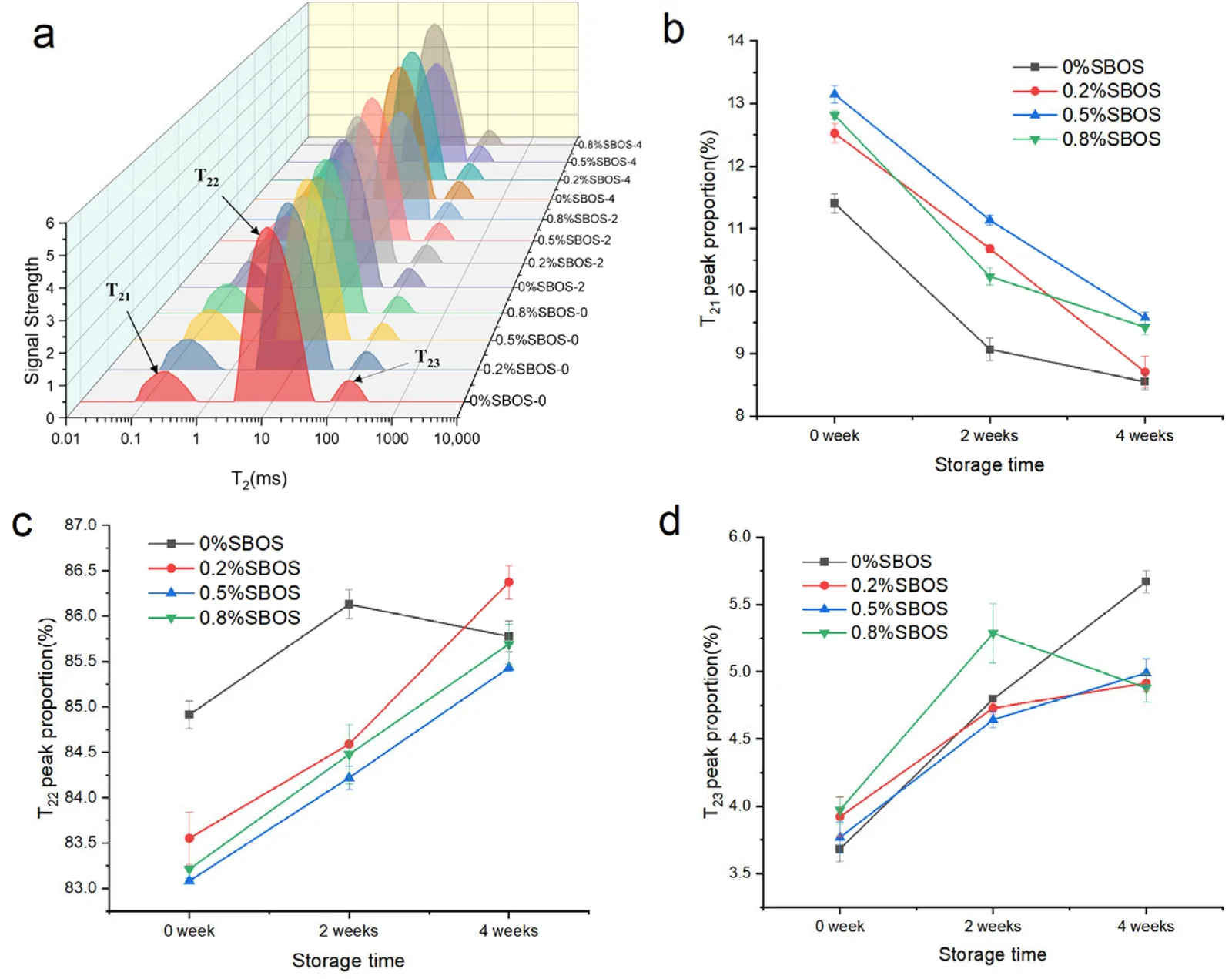
Changes in the NMR spin–spin relaxation (T_2_) with different SBOS ((**a**): T_2_ distribution curve; (**b**): T_21_ peak area proportions; (**c**): T_22_ peak area proportions; (**d**): T_23_ peak area proportions).

The transverse relaxation time (T_2_) measured by LF-NMR technology can characterize the mobility of water in dough, with a smaller T_2_ indicating lower mobility of water molecules. The T_2_ characteristic curve in Figure 3a typically has three peaks, namely T_21_, T_22_, and T_23_. T_21_ represents strongly bound water that is deeply bound to gluten, which is less likely to be lost during dough freezing. T_22_ represents weakly bound water, which can partially migrate to form ice crystals during freezing. T_23_ represents free water, which freezes first to form ice crystals when the dough is exposed to sub-zero temperatures [40,41]. By calculating the peak areas (T_21_, T_22_, T_23_) of water in different states based on the distribution diagram, it was found that the addition of SBOS altered the water state of fresh dough, with a decrease in strongly bound water content and increases in weakly bound water and free water content. Figure 3b, Figure 3c and Figure 3d show the peak areas of T_21_, T_22_, and T_23_, respectively, after freezing. After 4 weeks of frozen storage, the content of strongly bound water in the 0%SBOS dough decreased significantly, while the content of free water increased significantly. After 4 weeks of frozen storage, the T_21_ values (strongly bound water) of the 0.2%SBOS, 0.5%SBOS, and 0.8%SBOS dough groups were 1.87%, 11.93%, and 10.29% higher than those of the 0%SBOS dough, respectively. T_23_ (free water) decreased by 13.22%, 11.99%, and 13.93%, respectively, compared with the 0%SBOS dough. This indicates that dough supplemented with SBOS is effective in inhibiting the transformation of bound water into free water [29]. Further comparison of the moisture status of dough in the SBOS groups revealed that the addition of 0.8%SBOS significantly increased the content of weakly bound water and reduced the proportion of free water (*p* < 0.05).

When antifreeze agent SBOS is incorporated into frozen dough, it effectively inhibits the transformation of bound water into weakly bound water during the freezing and frozen storage process. SBOS molecules can interact with water molecules and gluten network components through hydrogen bonding and hydrophilic interactions, thereby enhancing the water-holding capacity of the dough system and stabilizing the state of tightly bound water. This stabilization reduces the amount of freezable water available for ice nucleation and crystal growth. With less free water participating in the phase transition to ice, the formation and excessive growth of ice crystals are significantly suppressed. These findings suggest that the cryoprotective effect of SBOS may be attributed to its ability to modulate water distribution during frozen storage. The addition of SBOS likely promotes the retention of bound water and inhibits the conversion to free water, which is consistent with the hypothesis that oligosaccharides can interact with water molecules through hydrogen bonding [42]. This interaction may reduce the amount of freezable water available for ice crystal formation, thereby minimizing mechanical damage to the gluten network. However, it is important to note that direct evidence of SBOS-water interactions was not obtained in this study, and this interpretation should be considered as a hypothesis that warrants further investigation.

### 3.4. Effect of Different Proportions of SBOS on the Secondary Structure of Frozen Dough

**Figure 4:**
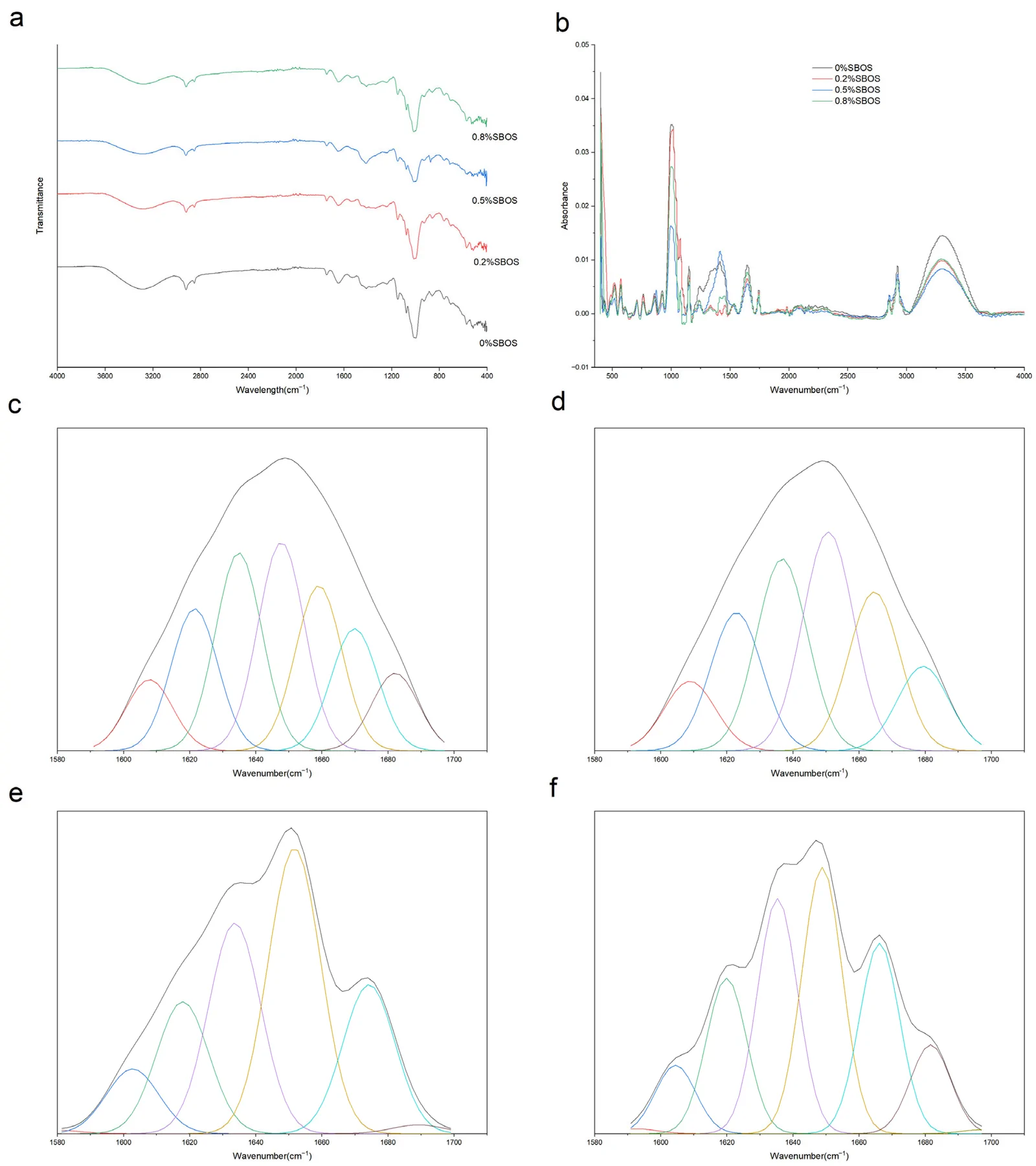
Original (**a**), deconvoluted (**b**) and 1600–1700 cm^−1^ FTIR spetra ((**c**): 0%SBOS, (**d**): 0.2%SBOS, (**e**): 0.5%SBOS, (**f**): 0.8%SBOS after storage for four weeks).

To further investigate the alterations in protein architecture, FTIR spectra were recorded for dough samples with or without SBOS supplementation (Figure 4). Subsequent data manipulation was performed using OMNIC 9 and PeakFit V4.12. The amide I region (about 1600–1700 cm^−1^) was extracted and processed through baseline correction (multi-point linear baseline), Gaussian smoothing, and second-derivative analysis to identify component peak positions. Curve deconvolution was performed using Gaussian peak fitting with the Levenberg–Marquardt algorithm, and the goodness of fit was assessed by R^2^ values. The deconvoluted peaks were assigned to specific secondary structures based on established literature references [43], enabling quantitative determination of the relative abundance of each structural component. Figure 4 presents both the original spectra (Figure 4a) and the deconvoluted and fitted spectra (Figure 4b–f) to allow assessment of the deconvolution quality.

Specifically, the amide I band (1600–1700 cm^−1^) of wheat proteins served as the most informative region for evaluating modifications in secondary structure composition. The absorption peak areas at 1650–1660 cm^−1^, 1610–1640 cm^−1^, 1660–1700 cm^−1^, and the region of 1640–1650 cm^−1^ were assigned to α-helix, β-sheet, β-turn, and random coil structures, respectively Jin et al. [40,44]. The relative proportions of these secondary structural elements were quantitatively calculated using PeakFit V4.12 (Cabit Information Technology Co., Ltd., Shanghai, China) (Table 1).

As the frozen storage duration extended from zero to four weeks, a consistent trend emerged across all dough samples: the α-helix content decreased whereas β-sheet content increased markedly. Conversely, the β-turn and random coil conformations of wheat proteins remained relatively stable throughout the frozen storage period. For the 0%SBOS group, α-helix content declined from 42.39% to 40.56%, accompanied by an increase in β-sheet content from 15.35% to 17.91% after 4 weeks of frozen storage. These observations align closely with previous investigations, demonstrating that α-helix structures are particularly susceptible to frozen storage and may undergo partial transformation into β-sheet configurations during freezing [35].

Upon SBOS incorporation, the reduction in α-helix content was substantially attenuated. Notably, at 0.5% SBOS addition, α-helix values were 43.98% and 43.54% at week 0 and week 2, respectively, exhibiting no statistically significant difference. This suggests that SBOS supplementation can effectively mitigate the conversion of α-helix to alternative secondary structures [45]. Another study by Zhu et al. [46] indicated that conformational alterations could unveil hydrophobic regions within the protein structure, leading to the release of bound water and its subsequent transformation into freezable water. This protective effect may be attributed to the inhibitory action of SBOS on ice recrystallization, thereby minimizing damage to disulfide bonds and hydrogen bonds within gluten proteins and consequently preventing the structural transition of α-helix [29]. Such stabilization of protein secondary structure contributes to enhanced elasticity and viscosity in dough systems. However, when SBOS concentration was elevated to 0.8%, the protective effect against α-helix reduction became less pronounced, indicating that beyond this threshold, increased SBOS dosage fails to confer additional cryoprotective benefits.

Regarding β-sheet structures, significant variations were observed across different SBOS concentrations as freezing time progressed, precluding definitive conclusions about the specific ameliorative effects of SBOS on frozen dough performance. Meanwhile, minimal changes were detected in both random coil and β-turn contents, a finding consistent with the observations reported by Jin et al. [40]. Collectively, these results demonstrate that SBOS incorporation can effectively stabilize the secondary structure of gluten proteins in dough, though optimization of the additive concentration represents a critical consideration for practical application.

### 3.5. Scanning Electron Microscopy (SEM)

The reticulated gluten network develops during dough kneading, while the supramolecular architecture of proteins governs numerous critical attributes of dough-based products, including texture, specific volume, and digestive properties [47]. Consequently, scanning electron microscopy (SEM) was employed to monitor microstructural alterations in dough throughout frozen storage. Frozen dough should exhibit a compact, continuous gluten network with starch granules evenly distributed throughout the structure.

**Figure 5:**
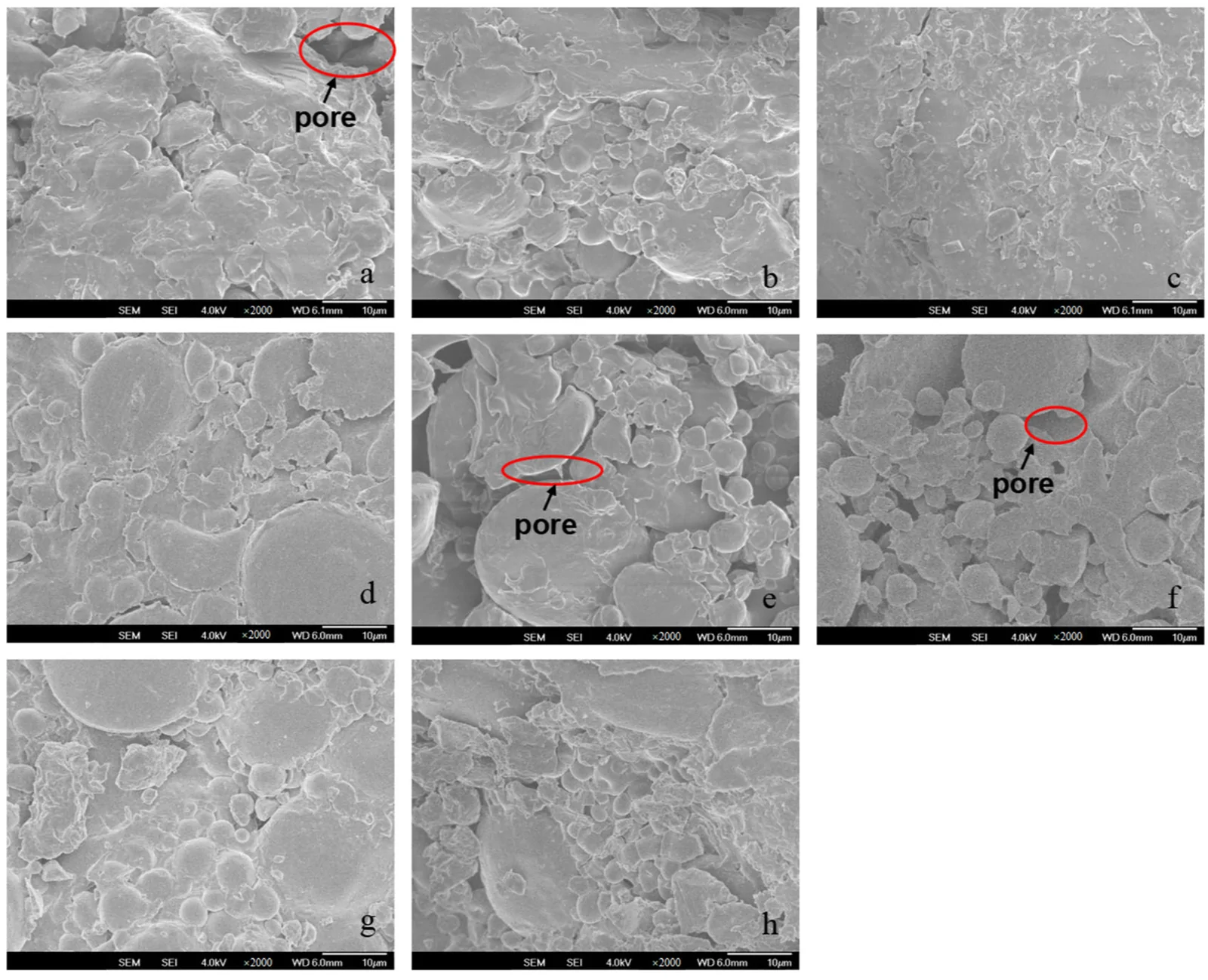
Microstructure of frozen dough containing SBOS at different storage periods ((**a**–**d**): 0%SBOS, 0.2% SBOS, 0.5% SBOS, 0.8% SBOS at 0-week storage; (**e**–**h**): 0%SBOS, 0.2% SBOS, 0.5% SBOS, 0.8% SBOS at 4 weeks storage).

As shown in Figure 5, following four weeks of frozen storage, substantial deterioration was evident in the 0%SBOS group (Figure 5a,e) without SBOS supplementation. The gluten matrix appeared coarse and fragmented, characterized by numerous irregularly distributed large pores. Starch granules were visibly detached from the protein network, indicative of compromised protein hydration, drastic gluten contraction, and compressive deformation of starch granules. These deteriorative changes can be primarily attributed to ice recrystallization and moisture redistribution phenomena [48].

Upon SBOS incorporation, frozen dough samples demonstrated markedly preserved gluten networks. Specifically, samples C, F, G, and H all maintained dense and continuous gluten structures, with sample C exhibiting the most favorable microstructural morphology, where starch granules remained uniformly integrated within the protein matrix. This protective effect stems from the inhibitory action of SBOS on moisture redistribution (Section 3.2), which constrains ice crystal growth and alleviates mechanical damage to the gluten framework.

Consequently, starch-gluten separation was substantially minimized, clearly demonstrating that SBOS addition confers significant protection to the gluten network in frozen dough systems. This finding is consistent with Tan et al. [31]’s observation that the improver enhances the microstructure of frozen dough, particularly by stabilizing the gluten network and promoting uniform starch distribution. However, when SBOS concentration was elevated to 0.8%, the protective efficacy showed no further improvement compared with lower dosages, suggesting that an optimal concentration range exists for maximizing cryoprotective benefits.

### 3.6. Specific Volume of Bread

**Figure 6:**
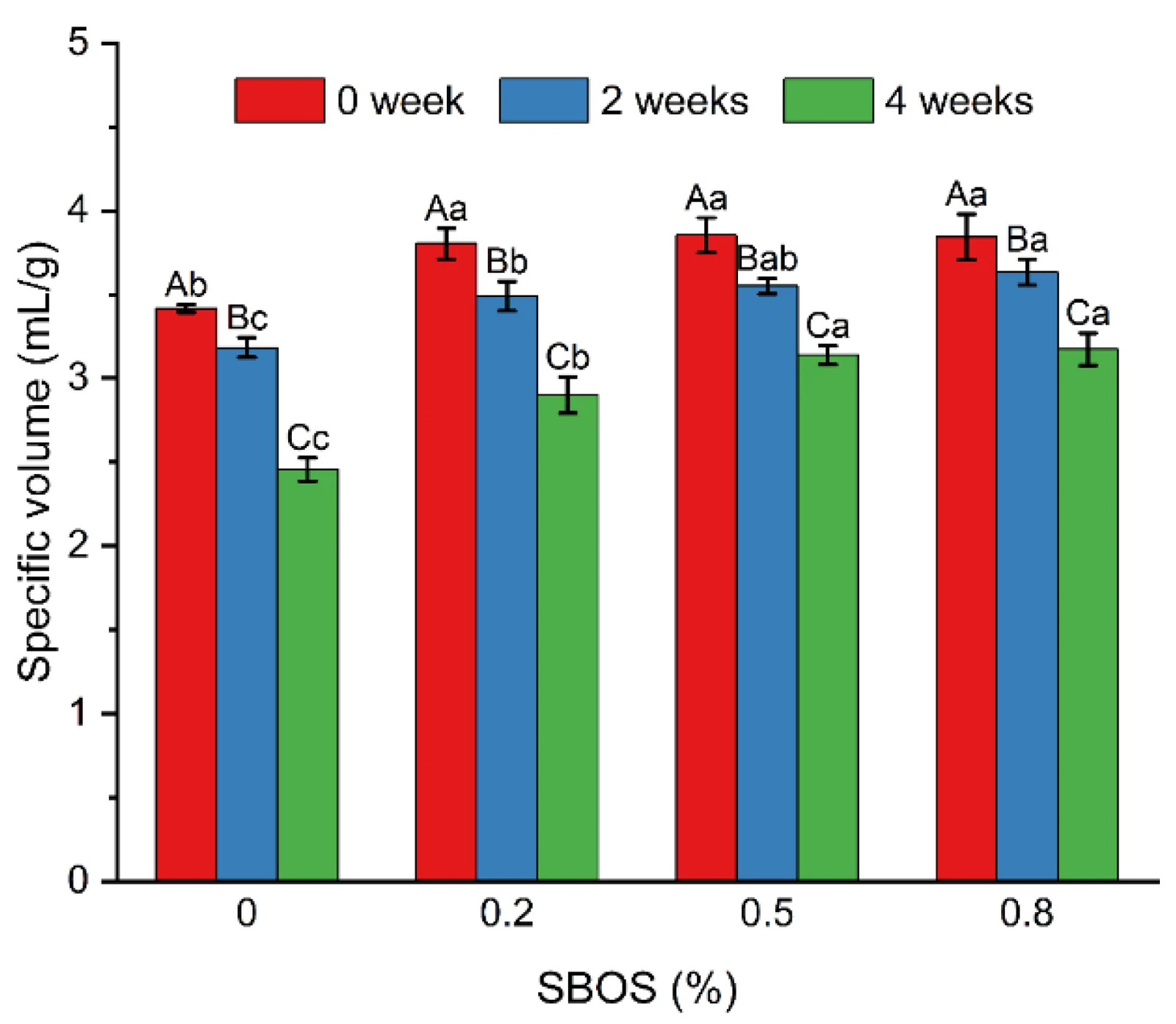
Effects of different SBOS ratios on specific volume of bread (Lowercase letters indicate significant differences among concentrations at the same freezing time *p* < 0.05; capital letters indicate significant differences among freezing times at the same concentration, *p* < 0.05).

Bread volume serves as one of the critical indicators for evaluating baking quality, directly reflecting the gas production capacity and gas retention properties of dough, thereby quantifying its baking characteristics [49]. As illustrated in Figure 6, prolonged frozen storage of dough resulted in a significant decline in specific bread volume, indicating cumulative detrimental effects on dough fermentation and baking performance. Specifically, a slight reduction in specific volume was observed after 2 weeks of frozen storage, whereas a marked decrease occurred at week 4. This deterioration was also visually apparent in the appearance of the finished bread products, suggesting that extended frozen storage severely compromises the structural integrity and gas-holding capability of the dough matrix.

SBOS addition exhibited a dose-dependent effect in mitigating frozen storage damage. While SBOS could not completely prevent the volume reduction after 2 and 4 weeks of storage, it did produce dough with significantly higher specific volumes compared with 0%SBOS at all measuring weeks (week 0, 2 and 4). At week 2, bread specific volume increased significantly with SBOS concentration (0.2%, 0.5%, and 0.8%), all outperforming the 0%SBOS, though no significant differences existed among dosages. This suggests a threshold effect: low-dose supplementation already enhances volume substantially, while higher doses confer no additional benefits, likely due to functional saturation of SBOS in the dough system. After 4 weeks of frozen storage, although bread volume declined across all groups, SBOS-supplemented samples consistently maintained higher volumes than the 0%SBOS. This indicates that SBOS imparts freeze–thaw stability to dough, delaying quality deterioration under prolonged storage, though it cannot fully prevent damage from extreme frozen stress. Overall, SBOS effectively improves the baking quality of frozen dough; however, its optimal dosage warrants further optimization considering cost-effectiveness and long-term stability.

As illustrated in Figure 6, prolonged frozen storage of dough resulted in a significant decline in specific bread volume, indicating cumulative detrimental effects on dough fermentation and baking performance. Specifically, a slight reduction in specific volume was observed after 2 weeks of frozen storage, whereas a marked decrease occurred at week 4. This deterioration was also visually apparent in the appearance of the finished bread products, which exhibited reduced height, compact crumb structure, and irregular surface characteristics, suggesting that extended frozen storage severely compromises the structural integrity and gas-holding capability of the dough matrix.

### 3.7. Textural Properties of Bread

TPA serves as an essential objective tool for evaluating bread quality and palatability, providing quantitative insights into the mechanical properties that govern sensory perception. High-quality bread is characterized by multiple attributes, including increased loaf volume, reduced crumb hardness, and uniform internal texture, rendering TPA parameters particularly suitable for assessing textural modifications in bread systems. Among these parameters, hardness represents a critical quality indicator that significantly influences consumer acceptance, as it directly correlates with the perceived freshness and overall eating experience of bread products [50].

The effects of SBOS addition on the textural properties of bread during frozen storage are presented in Table 2. Compared with the 0%SBOS group without SBOS supplementation, bread samples fortified with 0.2%, 0.5%, and 0.8% SBOS exhibited significant reductions in hardness (*p* < 0.05) at week 0, with decreases of 7.88%, 10.78%, and 13.27%, respectively. This phenomenon can be attributed to the cryoprotective functionality of SBOS during the freezing process. The incorporation of SBOS likely modulates ice crystallization kinetics, promoting the formation of smaller and more uniformly distributed ice crystals within the frozen dough matrix (Section 3.2). Consequently, the mechanical damage to the gluten network structure induced by ice crystal growth is substantially mitigated, thereby preserving the structural integrity and softness of the final bread crumb. Notably, this protective effect of SBOS was consistently observed at week 2 and week 4 of frozen storage, with the most pronounced reduction in hardness (15.27% decrease compared with the 0%SBOS) observed in the 0.5% SBOS group in week 4. These findings suggest that the beneficial impact of SBOS on bread hardness becomes increasingly prominent with extended frozen storage duration, although no statistically significant differences were detected among the various SBOS concentrations tested.

The chewiness profiles of bread samples followed patterns analogous to those observed for hardness, which is the same with Duan et al. [51] given that chewiness represents a derived parameter incorporating hardness, cohesiveness, and springiness values. Springiness, defined as the ability of bread crumb to recover its original shape and dimensions following the first compression cycle, serves as an indicator of the elastic component of the bread matrix. Cohesiveness, conversely, quantifies the internal structural integrity and resistance to disintegration of the bread crumb during compression, reflecting the degree of intermolecular bonding within the starch-protein network. Resilience, representing the instantaneous elastic recovery of the sample upon deformation, provides additional information regarding the viscoelastic behavior of the bread system [32]. At the same storage time, SBOS-treated samples exhibited significant changes in springiness, cohesiveness, and resilience compared with the 0%SBOS (significant differences are indicated by lowercase letters in Table 2). However, no significant differences were observed among the different SBOS addition levels (0.2%, 0.5%, and 0.8%). These results suggest that SBOS addition was effective, yet its beneficial effects did not improve with increasing dosage. Therefore, these three parameters support the efficacy of SBOS addition but do not influence the specific selection among 0.2%, 0.5%, and 0.8% SBOS supplementation levels in flour. Unlike hardness, these improvements remained stable throughout storage, suggesting these parameters are governed by similar structural mechanisms. Although ice recrystallization progressively damaged gluten network integrity in all samples, SBOS conferred partial protection, maintaining superior textural attributes against 0%SBOS.

### 3.8. Internal Texture Structure of Bread

**Figure 7:**
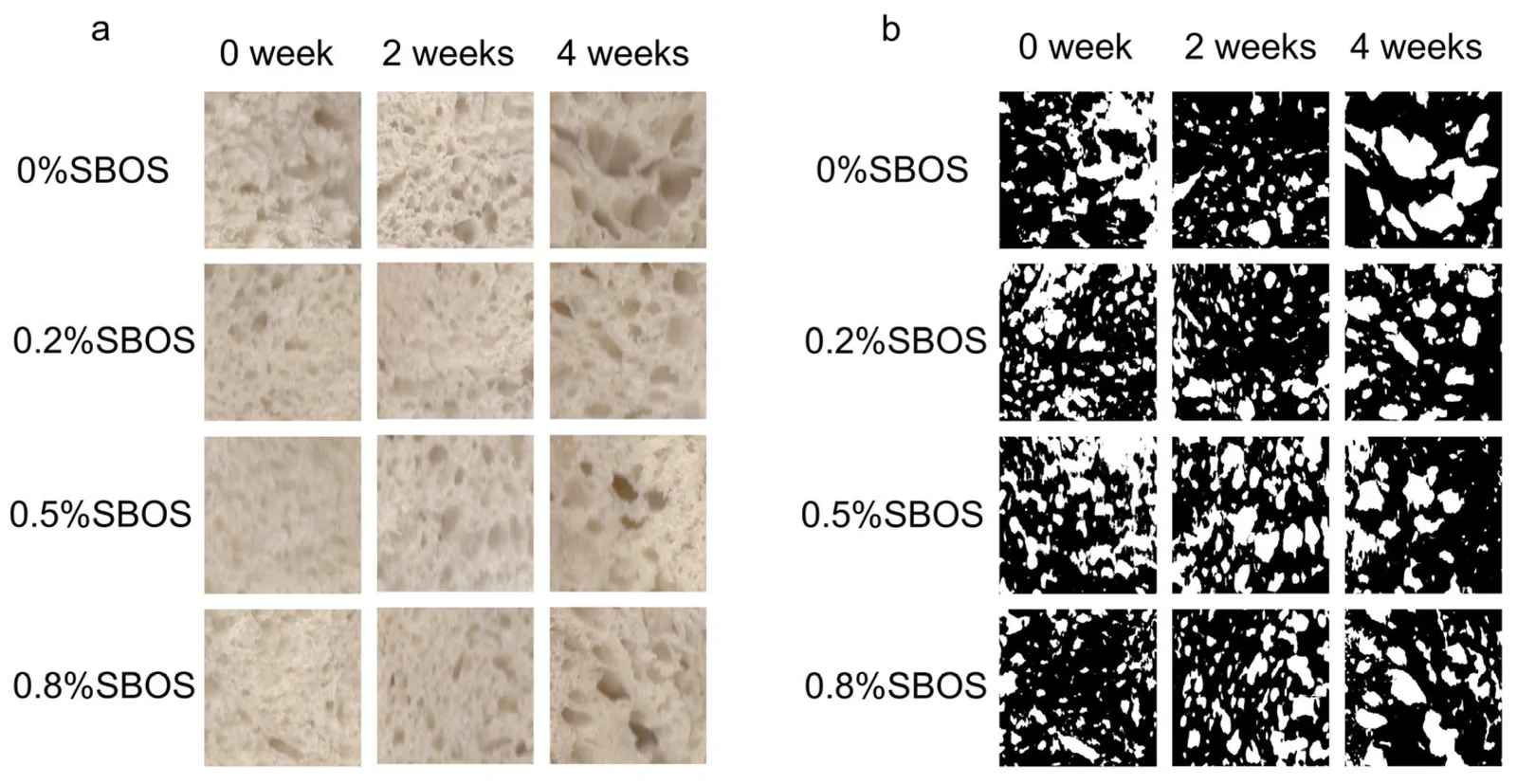
Bread texture structure ((**a**)-Cross-sectional scans of bread at different freezing durations; (**b**)-Image processed using ImageJ software).

Figure 7a illustrates the pore structure of bread prepared from frozen dough, providing a visual representation of the cross-sectional texture. In Figure 7b, the bright regions correspond to gas cells within the bread matrix, while the darker areas represent the cell walls. The density of these pores reflects the overall porosity of the bread. Table 3 presents pore density and mean cell size measurements. A higher pore density coupled with a lower mean cell size indicates a more uniform pore distribution, which correlates with improved elasticity and softness of the bread crumb [52].

Combined analysis of Figure 7 and Table 3 reveals that the 0%SBOS bread exhibited high pore density at week 0 of frozen storage, indicating a greater number of pores per unit area. However, after 4 weeks of frozen storage, the 0%SBOS bread showed a significant decline in pore density. Notably, SBOS supplementation significantly ameliorated this deterioration compared with the untreated 0%SBOS. Interestingly, SBOS bread did show higher pore density and smaller cell size compared with bread without SBOS, implying a significant improvement in bread texture structure when SBOS was added. Although no significant differences were observed in either stomatal density or mean pore area among bread supplemented with 0.2%, 0.5%, and 0.8% SBOS, this finding is still positive as it suggests that even at low concentrations, SBOS is able to exert a pronounced beneficial effect on the cross-sectional texture structure of bread.

### 3.9. Correlation Analysis

**Figure 8:**
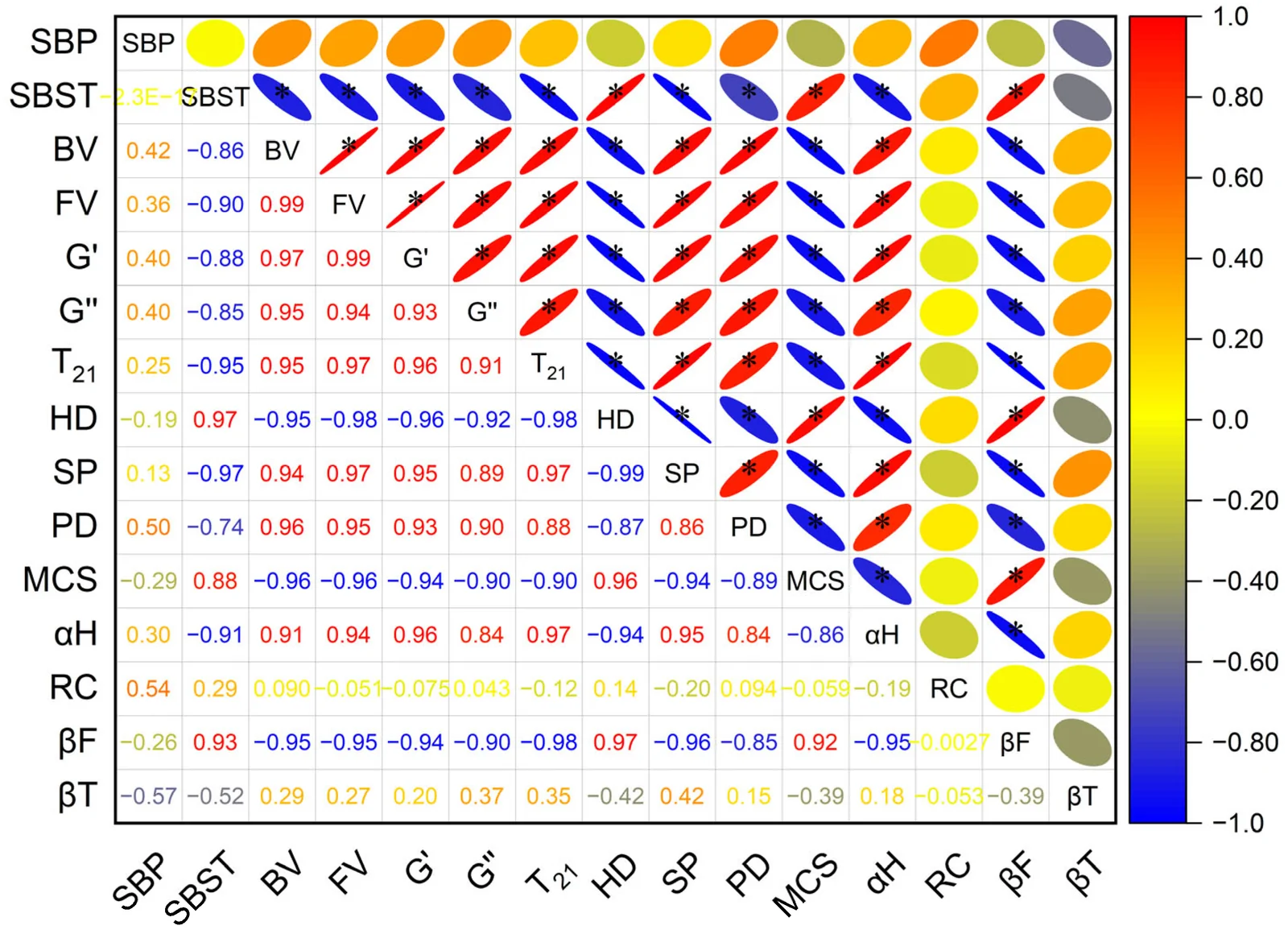
Correlation analysis of quality attributes and parameters (SBP: SBOS Proportion; SBST: SBOS storage time; BV: Bread specific volume; FV: Fermentation volume; HD: Hardness; SP: Springiness; PD: Pore density; Stomatal density; MCS: Mean cell size; αH: α-helix; RC: Random curling; βF: β-folding; βT: β-turning angle; symbols * denote statistical significance when *p* < 0.05).

To further investigate the underlying mechanisms of SBOS in frozen dough systems and elucidate the intrinsic relationships between frozen dough parameters and final bread quality, Pearson correlation analysis was conducted. As shown in Figure 8, no significant correlations were observed between SBP and any of the measured parameters, indicating that SBOS concentration was not linearly associated with quality attributes. However, this finding does not contradict the substantial improvements in frozen dough and bread quality demonstrated in the preceding sections of this study. Rather, it suggests that excessive SBOS addition may not confer additional benefits (0.8% or higher), and optimal quality enhancement can be achieved with moderate supplementation levels (0.2% and 0.5%). This observation provides practical guidance for controlling SBOS dosage in industrial production.

In contrast, SBST exhibited significant negative correlations with BV, FV, G′, G″, T_21_, SP, PD, and αH (*p* < 0.05), indicating that prolonged frozen storage significantly decreased bread specific volume, fermentation volume, storage modulus (G′), loss modulus (G″), strongly bound water content, springiness, pore density, and α-helix content. Conversely, bread hardness, mean cell size, and β-sheet content in frozen dough increased significantly. These results suggest that water migration occurred during frozen storage, and the increased bound water content effectively reduced the amount of freezable water, thereby alleviating freeze-induced damage to the dough matrix. Furthermore, the formation of a more compact and stable gluten network enhanced the rheological properties of frozen dough and improved the textural characteristics of bread products, which is consistent with the findings reported by Zhang et al. [32].

## 4. Conclusions

This study demonstrated that soybean oligosaccharides (SBOS) effectively alleviated freeze-induced deterioration of frozen dough during frozen storage, with 0.5% (*w*/*w* flour basis) showing the best overall performance under the conditions investigated in this study. SBOS reduced water migration by maintaining a higher proportion of bound water, preserved gluten network integrity, slowed the deterioration of rheological properties, and maintained the secondary structure of gluten proteins, thereby improving the stability of frozen dough during storage.

More importantly, these improvements in dough quality were reflected in the quality of the final bread. Compared with the control, breads prepared from SBOS-treated frozen dough exhibited a higher specific volume, softer crumb texture, and a more uniform crumb pore structure after frozen storage, indicating that SBOS effectively mitigated the adverse effects of freezing on bread quality. These results demonstrate that maintaining dough structure during frozen storage is critical for producing bread with desirable baking performance and textural characteristics.

Overall, SBOS can serve as an effective natural cryoprotectant for frozen dough and provides a promising clean-label strategy for improving both frozen dough stability and bread quality. The findings contribute to a better understanding of the cryoprotective role of soybean oligosaccharides and provide theoretical support for their application in frozen bakery products. Future studies should further investigate the molecular interactions between SBOS and gluten proteins, validate its cryoprotective mechanism under industrial frozen dough processing conditions, and evaluate its applicability in different bread formulations.

## Figures and Tables

**Table 1:** Variations in the secondary structure of gluten proteins in dough following the addition of SBOS.

	SBOS (%)	0 Week	2 Weeks	4 Weeks
**α-Helix (%)**	0	42.39 ± 0.39 ^Ab^	41.10 ± 0.25 ^Bc^	40.56 ± 0.33 ^Bb^
	0.2	42.54 ± 0.47 ^Ab^	41.83 ± 0.45 ^Ab^	40.77 ± 0.19 ^Bab^
	0.5	43.98 ± 0.08 ^Aa^	43.54 ± 0.58 ^Aa^	41.25 ± 0.10 ^Ba^
	0.8	43.59 ± 0.05 ^Aa^	42.35 ± 0.15 ^Bb^	40.93 ± 0.33 ^Cab^
**Random Curling (%)**	0	21.35 ± 0.19 ^Ba^	22.05 ± 0.41 ^Aa^	21.14 ± 0.11 ^Bb^
	0.2	21.43 ± 0.19 ^Aa^	21.11 ± 0.04 ^Ac^	21.17 ± 0.53 ^Ab^
	0.5	21.35 ± 0.70 ^Aa^	21.79 ± 0.09 ^Aab^	21.78 ± 0.33 ^Aab^
	0.8	21.25 ± 0.08 ^Ba^	21.46 ± 0.04 ^Bbc^	21.88 ± 0.18 ^Aa^
**β-Folding (%)**	0	15.35 ± 0.07 ^Ca^	16.38 ± 0.04 ^Ba^	17.91 ± 0.18 ^Aa^
	0.2	14.99 ± 0.64 ^Cab^	16.22 ± 0.17 ^Ba^	17.83 ± 0.45 ^Aa^
	0.5	14.40 ± 0.14 ^Cb^	15.16 ± 0.08 ^Bb^	16.53 ± 0.06 ^Ab^
	0.8	14.88 ± 0.53 ^Aab^	16.29 ± 0.04 ^Ba^	16.99 ± 0.15 ^Cc^
**β-Turning Angle (%)**	0	20.92 ± 0.64 ^Aa^	20.47 ± 0.48 ^Aab^	20.39 ± 0.27 ^Aa^
	0.2	21.04 ± 0.53 ^Aa^	20.84 ± 0.27 ^Aa^	20.23 ± 0.93 ^Aa^
	0.5	20.28 ± 0.63 ^Aa^	19.51 ± 0.54 ^Ac^	20.44 ± 0.42 ^Aa^
	0.8	20.28 ± 0.50 ^Aa^	19.91 ± 0.15 ^Abc^	20.20 ± 0.52 ^Aa^

Note: Lowercase letters indicate significant differences among concentrations at the same freezing time (*p* < 0.05); capital letters indicate significant differences among freezing times at the same concentration (*p* < 0.05).

**Table 2:** Hardness, chewiness, springiness, cohesiveness, resilience of bread made from frozen dough.

	SBOS (%)	0 Week	2 Weeks	4 Weeks
Hardness (g)	0	569.65 ± 15.95 ^Ca^	761.85 ± 7.94 ^Ba^	1005.39 ± 11.35 ^Aa^
	0.2	524.76 ± 5.74 ^Cb^	642.64 ± 14.35 ^Bc^	877.58 ± 11.08 ^Ab^
	0.5	508.22 ± 4.89 ^Cbc^	668.08 ± 2.59 ^Bb^	851.91 ± 3.02 ^Ac^
	0.8	494.08 ± 14.43 ^Cc^	680.53 ± 2.11 ^Bb^	864.2 ± 10.81 ^Abc^
Chewiness (g)	0	568.55 ± 6.93 ^Ca^	762.49 ± 9.85 ^Ba^	1004.31 ± 5.05 ^Aa^
	0.2	523.45 ± 4.44 ^Cb^	643.53 ± 7.59 ^Bd^	876.65 ± 24.67 ^Ab^
	0.5	508.92 ± 10.46 ^Cb^	712.93 ± 12.73 ^Bb^	851.69 ± 37.71 ^Ab^
	0.8	483.74 ± 10.86 ^Cc^	681.39 ± 2.14 ^Bc^	864.39 ± 12.55 ^Ab^
Springiness	0	0.92 ± 0.01 ^Ab^	0.84 ± 0.01 ^Bb^	0.79 ± 0.01 ^Cb^
	0.2	0.93 ± 0.01 ^Aa^	0.89 ± 0.01 ^Ba^	0.83 ± 0.02 ^Ca^
	0.5	0.95 ± 0.01 ^Aa^	0.89 ± 0.01 ^Ba^	0.83 ± 0.02 ^Ca^
	0.8	0.93 ± 0.01 ^Aa^	0.89 ± 0.01 ^Ba^	0.82 ± 0.01 ^Ca^
Cohesiveness	0	1.23 ± 0.02 ^Ab^	1.17 ± 0.01 ^Bb^	1.1 ± 0.01 ^Cb^
	0.2	1.30 ± 0.02 ^Aa^	1.27 ± 0.01 ^Ba^	1.16 ± 0.01 ^Ca^
	0.5	1.31 ± 0.02 ^Aa^	1.28 ± 0.01 ^Ba^	1.16 ± 0.01 ^Ca^
	0.8	1.32 ± 0.01 ^Aa^	1.28 ± 0.01 ^Ba^	1.17 ± 0.01 ^Ca^
Resilience	0	0.40 ± 0.02 ^Aa^	0.37 ± 0.01 ^Bb^	0.31 ± 0.01 ^Cb^
	0.2	0.42 ± 0.01 ^Aa^	0.39 ± 0.01 ^Ba^	0.34 ± 0.01 ^Ca^
	0.5	0.42 ± 0.01 ^Aa^	0.38 ± 0.01 ^Bab^	0.35 ± 0.01 ^Ca^
	0.8	0.42 ± 0.01 ^Aa^	0.38 ± 0.01 ^Bab^	0.35 ± 0.01 ^Ca^

Note: Springiness, cohesiveness, and resilience are dimensionless parameters. Lowercase letters indicate significant differences among concentrations at the same freezing time (*p* < 0.05); capital letters indicate significant differences among freezing times at the same concentration (*p* < 0.05).

**Table 3:** Effect of different SBOS ratios on the texture structure of bread pores.

	SBOS (%)	0 Week	2 Weeks	4 Weeks
Pore density (pores/cm^2^)	0	31.15 ± 2.40 ^Ab^	29.41 ± 3.24 ^Ab^	15.94 ± 2.31 ^Bb^
	0.2	46.73 ± 3.28 ^Aa^	39.09 ± 1.53 ^Ba^	30.93 ± 4.49 ^Ca^
	0.5	47.17 ± 4.99 ^Aa^	38.87 ± 2.22 ^Ba^	31.81 ± 2.77 ^Ca^
	0.8	46.22 ± 5.02 ^Aa^	37.92 ± 1.88 ^Ba^	33.05 ± 3.89 ^Ca^
Mean cell size (mm^2^)	0	0.93 ± 0.06 ^Ca^	1.27 ± 0.21 ^Ba^	1.9 ± 0.15 ^Aa^
	0.2	0.90 ± 0.00 ^Ba^	1.00 ± 0.00 ^Bb^	1.4 ± 0.12 ^Ab^
	0.5	0.90 ± 0.00 ^Ba^	1.00 ± 0.00 ^Bb^	1.3 ± 0.17 ^Ab^
	0.8	0.90 ± 0.00 ^Ba^	1.03 ± 0.06 ^Bb^	1.4 ± 0.21 ^Ab^

Note: Lowercase letters indicate significant differences among concentrations at the same freezing time (*p* < 0.05); capital letters indicate significant differences among freezing times at the same concentration (*p* < 0.05).

## Data Availability

The original contributions presented in this study are included in the article; further inquiries can be directed to the corresponding authors.
